# Sensory Alterations in Patients with Isolated Idiopathic Dystonia: An Exploratory Quantitative Sensory Testing Analysis

**DOI:** 10.3389/fneur.2017.00553

**Published:** 2017-10-17

**Authors:** Lejla Paracka, Florian Wegner, Christian Blahak, Mahmoud Abdallat, Assel Saryyeva, Dirk Dressler, Matthias Karst, Joachim K. Krauss

**Affiliations:** ^1^Department of Neurology, Hannover Medical School, Hannover, Germany; ^2^Center for Systems Neuroscience, Hannover, Germany; ^3^Faculty of Medicine Mannheim, Department of Neurology, University of Heidelberg, Mannheim, Germany; ^4^Department of Neurosurgery, Hannover Medical School, Hannover, Germany; ^5^Department of Anesthesiology, Hannover Medical School, Hannover, Germany

**Keywords:** dystonia, idiopathic, sensory system, alterations, quantitative sensory testing

## Abstract

Abnormalities in the somatosensory system are increasingly being recognized in patients with dystonia. The aim of this study was to investigate whether sensory abnormalities are confined to the dystonic body segments or whether there is a wider involvement in patients with idiopathic dystonia. For this purpose, we recruited 20 patients, 8 had generalized, 5 had segmental dystonia with upper extremity involvement, and 7 had cervical dystonia. In total, there were 13 patients with upper extremity involvement. We used Quantitative Sensory Testing (QST) at the back of the hand in all patients and at the shoulder in patients with cervical dystonia. The main finding on the hand QST was impaired cold detection threshold (CDT), dynamic mechanical allodynia (DMA), and thermal sensory limen (TSL). The alterations were present on both hands, but more pronounced on the side more affected with dystonia. Patients with cervical dystonia showed a reduced CDT and hot detection threshold (HDT), enhanced TSL and DMA at the back of the hand, whereas the shoulder QST only revealed increased cold pain threshold and DMA. In summary, QST clearly shows distinct sensory abnormalities in patients with idiopathic dystonia, which may also manifest in body regions without evident dystonia. Further studies with larger groups of dystonia patients are needed to prove the consistency of these findings.

## Introduction

The dystonias are a heterogenous group of movement disorders, which are defined by abnormal movements or postures (1, 2). According to the modern definition, dystonia represents a movement disorder, which is characterized by sustained or intermittent muscle contractions causing abnormal, often repetitive movements, postures, or both. Dystonic movements are typically patterned, twisting, and may be tremulous. Dystonia is often initiated or worsened by voluntary action and associated with overflow muscle activation (1).

Idiopathic and inherited dystonia has been regarded as a disorder of basal ganglia activity and associated circuits. The pathophysiology of dystonia is not fully clarified, but several mechanisms, such as decreased inhibition, altered plasticity, and dysfunction of oscillatory activity appear to be involved (3). In addition, there is limited evidence of involvement of sensory systems in the pathophysiology of dystonia. Both imaging and electrophysiological studies have revealed abnormalities in sensorimotor networks (4, 5) and also in cerebello-thalamo-cortical pathways (6, 7).

Patients with dystonia often complain about pain, although the clinical examination and neurophysiological testing remain normal (8). Further, they use certain maneuvers in order to temporarily improve dystonia. This phenomenon has been recognized as sensory trick (or geste antagoniste) suggesting a possible role of the sensory system in clinical manifestation of dystonia (9).

Other hints for involvement of sensory systems include abnormal sensory processing (10, 11) and altered temporal and spatial discrimination of tactile stimuli (12–16).

Hence, we investigated the somatosensory integrity in patients with idiopathic dystonia using quantitative sensory testing (QST) (17, 18). This method allows the investigation of different sensory qualities such as heat, cold, paradox sensations (the ability to differentiate warmth and cold), touch, pain, pressure, and vibrations, which are transmitted by Aβ, Aδ, and C fibers in the periphery. The aim of the study was to investigate whether sensory alterations are confined to the dystonic body segments or show wider involvement in patients with dystonia.

## Materials and Methods

### Subjects

Patients with idiopathic or inherited dystonia were considered for this study. Exclusion criteria were polyneuropathy, cognitive disturbances, or any other neurological disease. The patients who received botulinum toxin within 6 months prior to the testing were excluded from the study. It is known that the effect of botulinum toxin in the nociceptive perception may last longer than the effect on the muscle relaxation (19). The cut-off period of 6 months was chosen in order to avoid any long-lasting effect of the botulinum toxin on sensory perception. Twenty patients with isolated dystonia (10 men and 10 women, mean age ± SD 52.7 ± 18.5) and 19 age and sex matched controls (10 men and 9 women, mean age ± SD 49.1 ± 18.9) were included in the study. Eight patients had generalized dystonia, five segmental dystonia with upper extremity involvement, and seven had isolated cervical dystonia. Two patients had genetically proven DYT1 hereditary dystonia. Three of the seven patients with cervical dystonia reported shoulder or neck pain, otherwise, there were no pain symptoms or overt sensory deficits.

All patients and controls signed written informed consent. The ethics committee of Hannover Medical School approved the study (No. 6307).

### Neurophysiological and Clinical Assessment

In all patients, standard nerve conduction velocity measurements to assess large myelinated fibers was performed. Neurographies and nerve conduction velocities conducted from ulnar, radial, and sural nerves were normal in all instances. Somatosensory evoked potentials were performed to exclude central sensory pathway disturbance. The Burke–Fahn–Marsden Dystonia Rating Scale (BFM) was used in all patients and the Toronto Western Spasmodic Torticollis Rating Scale (TWSTRS) in patients with cervical dystonia. Hand and arm dystonia was scored using the BFM motor subscore for arm (0–16); the upper extremity with the higher score was determined as clinically more affected side, and the one with the lower score as clinically less affected. Because of the axial involvement in cervical dystonia, for the shoulders, the mean score of both sides was used for further analysis and comparison to controls.

### Quantitative Sensory Testing

The QST battery was applied according to the protocol of the German Research Network of Neuropathic Pain (18, 20). QST allows the assessment of specific myelinated Aδ- and unmyelinated C-fibers for temperature and pain, but also the evaluation of touch and vibration eliciting activity in large myelinated Aβ-fibers. It also allows testing of sensory modalities not assessable by conventional neurophysiological methods. QST uses various stimuli to investigate the different sensory modalities such as a thermal test, mechanical stimuli (pinprick set), stimuli for touch (Fray Hair filaments, cotton wool, Q-tip, brush), vibration (tuning fork), and pressure (pressure pain algometer).

The results of patients with upper extremity involvement in dystonia were compared to the side less affected by dystonia and to the normal values of healthy controls. In cervical dystonia, the mean of the QST measures of both shoulders were calculated and compared to those of the controls. In patients with cervical dystonia, assessment included also QST of the non-dystonic hands using the values of both hands for comparison with the corresponding values of the controls.

Twelve tests were performed as previously described (17). Shortly, thermal thresholds were measured with the thermal sensory analyzer (MEDOC, Ramat Yishari, Israel). Cold detection threshold (CDT) and hot detection threshold (HDT) were registered with decreasing or increasing temperature stimuli, respectively, that were applied with a thermode of 3 cm^2^. The baseline temperature of the thermode was 32°C and it increased and decreased the temperature by 1°C/s. For detection thresholds, subjects were asked to press the stop button in the moment they perceived the warm or cold stimuli. The same procedure was used for the thermal sensory limen (TSL), the difference threshold for alternating cool and warm stimuli (17). The TSL has been designed to test paradoxical sensations in heat perception and the capability to detect fast changes of temperature. Cold pain threshold and hot pain threshold (HPT) were measured by pressing a stop button when the sensation for cold or warm reached a painful perception (range 0–50°C).

The tactile detection threshold (TDT) was recorded with a set of Frey filaments (OptiHair MARSTOCKnervtest, Marburg, Germany), which exert forces of 0.25–512 mN. The patients had to indicate if they felt the stimuli with their eyes closed. The filaments with smaller forces were gradually introduced and the threshold was recorded where they lastly did not perceive any sensation. The final value was the geometrical mean of five series of ascending and descending stimuli (17).

The mechanical pain threshold (MPT) was registered with a set of weighted pinprick stimulators with a flat contact area of 0.25 mm^2^ diameter inducing pressure forces of 8, 16, 32, 64, 128, 256, and 512 mN (MRC Systems, Heidelberg, Germany). Using this set of instruments and escalating the stimulation force from 8 mN, subjects were asked to indicate the sharp sensation pain threshold. After determination of the sharp sensation pain threshold, the stimulation force was gradually reduced to determine the dull sensation threshold. The ascending and descending stimuli were applied five times and limits for dull and sharp sensations were registered. The MPT threshold was the geometrical mean of five series of ascending and descending stimuli (21).

The mechanical pain sensitivity to pain sensitivity for pinprick (PSP) and dynamic mechanical allodynia (DMA) were determined with a set of pinprick stimulators (MRC Systems, Heidelberg, Germany) and the following tactile stimulators: cotton wool (MEDIWOOD, megro GmbH, Wesel, Germany), a cotton Q-tip and a brush (Somedic, Hörby, Sweden). Sensitivity to sharp pin prick sensation and to tactile stimulators was determined. Dynamic mechanical allodynia represents pain due to light moving mechanical stimuli. Subjects were asked to estimate the pain elicited by the stimuli (pinprick and tactile stimulators) on an analogue scale from 0 to 100, whereby 0 represents no pain (touch and no sharp sensation) and 100 represents the most unbearable intense pain. Every sharp sensation had to be evaluated with a value larger than 0. These sharp sensations even when not percepted as painful had to be scored with a number above 0 on an analogue scale. The testing was performed with the eyes closed. The final value was the arithmetical mean of the ratings across all stimuli (21).

The wind up ratio (WUR) represents the perceptual correlate of temporal pain summation (17) induced by the frequency-dependent increase of action potentials of the spinal cord neurons evoked by stimulation of C-fibres (22). It was tested with the pinprick stimulator of 256 mN where single stimuli were compared to 10 pinprick stimuli of the same physical intensity (1/s applied within an area of 1 cm^2^). The testing was applied to five different skin areas (back of the hand or shoulder) and subjects were asked to rate the pain on an analogue scale from 0 to 100. The final WUR value was the ratio between the average sores of 5 series of 10 pinprick stimulations each and 5 single stimuli.

The vibration detection threshold (VT) was determined with a Rydel Seiffer fork (c64 Hz, 8/8 scale) placed at the processus styloideus ulnae or the acromion. Testing was repeated three times and the final value was the arithmetical mean of three sensations.

Finally, the pressure pain threshold (PPT), which represents the resistance of the muscles was recorded by pressure pain algometer (Somedic Algometer, Hörby, Sweden). This device allows quantification of the tenderness of muscles. It contains a rubber disc with an area of 1 cm^2^ that displays pressure on a specific part of the body. PPT was applied to the M. abductor pollicis brevis and M. trapezius (17).

### Neuropsychological Examination

Patients and controls underwent neuropsychlogical testing for cognition (Mini Mental Score) and attention (Trail Making Test) as such impairment may interfere with the results of the QST testing.

### Statistical Analysis

To determine the data distribution, the Shapiro–Wilk test and visualisation assessment were used. Normally distributed data were analyzed as raw data and with 95% confidence interval. Non-normally distributed data were log_10_ transformed in order to reach normalization. Test for normality was carried out in each sequence of the analysis and if log_10_ transformation was performed. To compare both extremity sides to the normal values throughout different QST parameters, *z*-transformation was used for each subject according to the formula *z*-score = (X_single patient_ − Mean_controls_)/SD_controls_. QST parameters for hand were first analyzed for all patients (*n* = 20), including patients with isolated cervical dystonia (*n* = 7). The mean score of both hands was compared to the mean score of controls. Next, the data of 13 patients with upper extremity involvement in dystonia were compared with controls in relation to the clinically more or less affected side. The mean score of both sides of the shoulder QST in patients with cervical dystonia was compared to the mean score of the shoulder QST of the controls. The same procedure was done in the hand QST for patients with cervical dystonia. *Z*-scores greater than 2 were considered as gain of sensory function and values below 2 were reported as loss of sensory function. Aside to *z*-scores, normally distributed data were analyzed by applying the unpaired student’s *t*-test without correction for multiple comparisons due to the exploratory nature of this study.

Correlation analysis between BFM and QST values of the hand and TWSTRS and QST values of the shoulder was performed with the Spearman’s Rho test. For statistical analyses and visualization of data, we used SPSS (IBM Deutschland, Ehningen, Germany). Two-sided *p*-values <0.05 were considered statistically significant.

## Results

The mean BFM motor score (±SD) of all patients was 16.4 ± 9.3 (generalized dystonia 28.3 ± 5.7, segmental 11.3 ± 4.1, and cervical 6.0 ± 2.2). A significant difference (*p* = 0.023) in the arm BFM subscore was evident when comparing the more affected upper extremity (7.5 ± 3.8) with the less affected upper extremity (3.7 ± 2.8) in patients with upper extremity involvement of dystonia (*n* = 13). The mean of the TWSTRS motor score (±SD) in patients with cervical dystonia was 8.6 ± 2.5 (*n* = 7).

**Figure 1:**
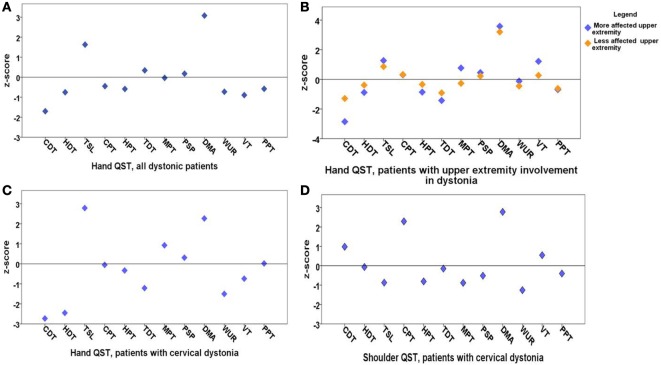
Means of *z*-scores of quantitative sensory testing (QST). **(A)** Hand QST in all dystonia patients (*n* = 20); **(B)** hand QST in patients with upper extremity involvement in dystonia (*n* = 13) after exclusion of cervical dystonia patients; there is no significant sensory difference between clinically more and less affected dystonic sides; **(C)** hand QST in patients with cervical dystonia (*n* = 7); **(D)** shoulder QST in patients with cervical dystonia (*n* = 7). QST parameters: cold detection threshold (CDT), hot detection threshold (HDT), thermal sensory limen (TSL), cold pain threshold (CPT), hot pain threshold (HPT), tactile detection threshold (TDT), mechanical pain threshold (MPT), mechanical pain sensitivity for pinprick (PSP), dynamic mechanical allodynia (DMA), wind up ratio (WUR), vibration threshold (VT), pressure pain threshold (PPT). *z*-scores higher than 2 are considered as sensory gain and *z*-scores below 2 are considered as sensory loss.

Quantitative sensory testing of the hand in the whole group of patients with dystonia (*n* = 20) showed a sensory gain for dynamic mechanic allodynia (*p* = 0.001) and thermal sensory limen (*p* = 0.04) and a tendency to sensory loss for cold detection threshold (*p* = 0.055) in comparison to matched controls (*n* = 20, Figure 1A; Table 1).

**Table 1:** Hand quantitative sensory testing (QST) of all patients with dystonia (*n* = 20) compared to matched healthy controls (*n* = 19) showing raw data and log transformed parameters of non-normally distributed data.

QST parameters	Patients with dystonia raw	Controls raw	Patients with dystonia log	Controls log	*p*-Values
CDT (°C)	−2.29 ± 1.6	−1.43 ± 0.51			0.055
HDT (°C)	3.63 ± 2.2	2.42 ± 1.61	0.49 ± 0.24	0.53 ± 0.06	n.s.
TSL (°C)	5.89 ± 3.08	3.56 ± 1.96	0.51 ± 0.19	0.32 ± 0.22	0.04
CPT (°C)	17.47 ± 9.19	13.61 ± 8.66			n.s.
HPT (°C)	42.51 ± 4.16	44.12 ± 2.55			n.s.
TDT (mN)	3.02 ± 2.45	1.26 ± 1.27	0.39 ± 0.31	0.31 ± 0.18	n.s
MPT (mN)	71.17 ± 107 ± 4	49.60 ± 32.88	1.56 ± 0.72	1.58 ± 0.36	n.s.
PSP	1.3 ± 1.2	1.15 ± 0.85	0.33 ± 0.18	0.31 ± 0.14	n.s.
DMA	0.13 ± 0.0627	0.01 ± 0.03	0.05 ± 0.02	0.004 ± 0.001	0.001
WUR (ratio)	2.64 ± 1.77	1.94 ± 0.26	0.34 ± 0.25	0.28 ± 0.06	n.s.
VDT (/8)	7.32 ± 0.58	7.75 ± 0.48	0.19 ± 1.16	0.08 ± 0.13	n.s.
PPT (kPa)	340.0 ± 81.7	367.6 ± 119.57			n.s.

*QST parameters are given as means ± SD. p-Values are based on raw data when the data are normally distributed and on log transformed data when they are non-normally distributed*.

*CDT, cold detection threshold; HDT, hot detection threshold; TSL, thermal sensory limen; CPT, cold pain threshold; HPT, hot pain threshold; TDT, tactile detection threshold; MPT, mechanical pain threshold; PSP, mechanical pain sensitivity for pinprick; DMA, dynamic mechanical allodynia; WUR, wind up ratio; VT, vibration threshold; PPT, pressure pain threshold*.

In the next step, patients with cervical dystonia were excluded in order to analyze more homogenously the relation of dystonia and sensory function. Compared to the control group, QST detected sensory gain for dynamic mechanical allodynia bilaterally (*p* = 0.001) and for thermal sensory limen on the more affected upper extremity with dystonia (*p* = 0.01). Additionally, a more limited sensory loss for cold detection threshold on the more affected upper extremity was revealed (*p* = 0.052) (*n* = 13, Figure 1B; Table 2). *z*-scores of the clinically more affected upper extremity showed a higher shift from baseline than the clinically less affected upper extremity in all instances compared to controls (Figure 1B).

**Table 2:** Hand quantitative sensory testing (QST) of patients with upper extremity involvement in dystonia (*n* = 13), after exclusion of patients with cervical dystonia, compared to matched healthy controls (*n* = 19).

QST parameter	Patients with dystonia clinically more affected upper extremity raw	Patients with dystonia clinically less affected upper extremity raw	Controls raw	Patients with dystonia clinically more affected upper extremity log	Patients with dystonia clinically less affected upper extremity log	Controls log	*p-*Values clinically more affected upper extremity	*p*-Values clinically less affected upper extremity
CDT (°C)	−2.69 ± 2.38	−2.31 ± 1.17	−1.43 ± 0.51	0.98 ± 0.06	0.94 ± 0.16	1.02 ± 0.02	0.052	n.s.
HDT (°C)	3.17 ± 1.66	3.17 ± 1.89	2.42 ± 1.61	1.62 ± 0.04	1.63 ± 0.05	1.64 ± 0.02	n.s.	n.s.
TSL (°C)	6.12 ± 2.46	6.02 ± 4.38	3.56 ± 1.96	0.75 ± 0.18	0.68 ± 0.31	0.51 ± 0.19	0.01	n.s.
CPT (°C)	16.22 ± 8.97	16.42 ± 8.09	13.61 ± 8.66				n.s.	n.s.
HPT (°C)	41.42 ± 3.88	43.14 ± 4.5	44.12 ± 2.55				n.s.	n.s.
TDT (mN)	15.45 ± 46.21	10.63 ± 32.73	1.26 ± 1.27	0.58 ± 0.32	0.44 ± 0.56	0.29 ± 0.2	n.s.	n.s.
MPT (mN)	74.82 ± 147.19	40.77 ± 36.08	49.6 ± 32.88	1.47 ± 0.54	1.42 ± 0.45	0.29 ± 0.36	n.s.	n.s.
PSP	1.61 ± 1.45	0.12 ± 0.82	1.15 ± 0.85	0.35 ± 0.24	0.33 ± 0.15	0.29 ± 0.18	n.s.	n.s.
DMA	0.14 ± 0.08	0.12 ± 0.05	0.01 ± 0.03	0.05 ± 0.02	0.05 ± 0.02	0.03 ± 0.02	0.001	0.001
WUR (ratio)	2.59 ± 1.67	2.7 ± 1.91	1.94 ± 0.26	0.33 ± 0.28	0.34 ± 0.29	0.31 ± 0.03	n.s.	n.s.
VDT (/8)	7.24 ± 0.68	7.54 ± 0.58	7.75 ± 0.48	0.22 ± 0.19	0.10 ± 0.15	0.05 ± 0.14	n.s.	n.s.
PPT (kPa)	348.67 ± 73.05	358.89 ± 103.51	367.6 ± 119.57	2.53 ± 0.09	2.54 ± 0.20	2.54 ± 0.20	n.s.	n.s.

*There is no significant sensory difference between patients’ clinically more affected and less affected arm/hand. QST parameters are shown as means ± SD. p-Values are based on raw data when the data are normally distributed and on log transformed data when they are non-normally distributed*.

*CDT, cold detection threshold; HDT, hot detection threshold; TSL, thermal sensory limen; CPT, cold pain threshold; HPT, hot pain threshold; TDT, tactile detection threshold; MPT, mechanical pain threshold; PSP, mechanical pain sensitivity for pinprick; DMA, dynamic mechanical allodynia; WUR, wind up ratio; VT, vibration threshold; PPT, pressure pain threshold*.

In the hand QST of the seven patients with isolated cervical dystonia, a sensory decrease for cold (*p* = 0.04) and hot detection thresholds (*p* = 0.03) was found as well as increased sensation for allodynia (*p* = 0.02) and to a lesser extent for thermal sensory limen (*p* = 0.06) compared to controls (*n* = 7, Figure 1C; Table 3).

**Table 3:** Hand quantitative sensory testing (QST) in patients with cervical dystonia (*n* = 7) compared to age-matched healthy controls (*n* = 9).

QST parameter	Cervical dystonia raw	Controls raw	Cervical dystonia log	Controls log	*p-*Values
CDT (°C)	−2.03 ± 1.08	−1.13 ± 0.33	0.44 ± 0.18	0.58 ± 0.03	0.04
HDT (°C)	4.17 ± 1.77	1.85 ± 0.54	0.58 ± 0.21	0.28 ± 0.148	0.03
TSL (°C)	5.65 ± 2.21	2.89 ± 0.99			0.06
CPT (°C)	17.48 ± 10.61	17.91 ± 8.97			n.s.
HPT (°C)	42.29 ± 4.02	43.33 ± 3.14			n.s.
TDT (mN)	4.13 ± 7.61	2.76 ± 1.41	0.44 ± 0.43	0.23 ± 0.11	n.s.
MPT (mN)	77.49 ± 57.35	44.77 ± 35.21	1.78 ± 0.36	1.51 ± 0.39	n.s.
MPS	1.58 ± 1.83	1.24 ± 1.08	0.344 ± 0.261	0.313 ± 0.183	n.s.
DMA	0.13 ± 0.07	0.024 ± 0.052	0.052 ± 0.026	0.01 ± 0.022	0.02
WUR (ratio)	2.21 ± 0.91	1.91 ± 0.19	0.49 ± 0.12	0.46 ± 0.02	n.s.
VDT (/8)	7.13 ± 0.29	758 ± 0.61			n.s.
PPT (kPa)	330 ± 86.38	327.87 ± 139.38			n.s.

*The QST values represent the mean of both hands. QST parameters are given as means ± SD. p-Values are based on raw data when the data are normally distributed and on log transformed data when they are non-normally distributed*.

*CDT, cold detection threshold; HDT, hot detection threshold; TSL, thermal sensory limen; CPT, cold pain threshold; HPT, hot pain threshold; TDT, tactile detection threshold; MPT, mechanical pain threshold; PSP, mechanical pain sensitivity for pinprick; DMA, dynamic mechanical allodynia; WUR, wind up ratio; VT, vibration threshold; PPT, pressure pain threshold*.

In the shoulder QST of the seven patients with cervical dystonia, a higher score for cold pain threshold (*p* = 0.008) and dynamic mechanical allodynia (*p* = 0.001) was detected in comparison to controls (*n* = 7, Figure 1D; Table 4).

**Table 4:** Shoulder quantitative sensory testing (QST) in patients with cervical dystonia (*n* = 7) compared to age-matched healthy controls (*n* = 9).

QST parameter	Cervical dystonia raw	Controls raw	Cervical dystonia log	Controls log	*p-*Values
CDT (°C)	−1.57 ± 0.81	−2.09 ± 0.33			n.s.
HDT (°C)	4.05 ± 1.47	4.21 ± 2.23	0.56 ± 0.17	0.58 ± 0.18	n.s.
TSL (°C)	5.51 ± 2.29	7.93 ± 4.34	0.71 ± 0.18	0.87 ± 0.19	n.s.
CPT (°C)	19.38 ± 8.75	9.27 ± 4.41			0.008
HPT (°C)	44.28 ± 4.38	45.91 ± 2.02			n.s.
TDT (mN)	5.35 ± 7.39	3.69 ± 3.93	0.53 ± 0.46	0.57 ± 0.29	n.s.
MPT (mN)	35.23 ± 32.03	45.42 ± 13.76	1.38 ± 0.41	1.64 ± 0.12	n.s.
PSP	0.63 ± 0.52	0.67 ± 0.12	−0.39 ± 0.33	−1.77 ± 0.07	n.s.
DMA	0.634 ± 0.055	0.03 ± 0.032	0.052 ± 0.021	0.01 ± 0.015	0.001
WUR (ratio)	2.09 ± 1.37	2.16 ± 0.28	0.26 ± 0.25	0.33 ± 0.06	n.s.
VDT (/8)	7.2 ± 0.44	6.92 ± 0.69			n.s.
PPT (kPa)	429.4 ± 132.32	470.75 ± 126.96			n.s.

*The QST values represent the mean of both shoulders. QST parameters are given as means ± SD. p-Values are based on raw data when the data are normally distributed and on log transformed data when they are non-normally distributed*.

*CDT, cold detection threshold; HDT, hot detection threshold; TSL, thermal sensory limen; CPT, cold pain threshold; HPT, hot pain threshold; TDT, tactile detection threshold; MPT, mechanical pain threshold; PSP, mechanical pain sensitivity for pinprick; DMA, dynamic mechanical allodynia; WUR, wind up ratio; VT, vibration threshold; PPT, pressure pain threshold*.

There was no significant correlation between the BFM motor score and any QST measure (Table 5). There was also no correlation between the TWSTRS motor score and QST measures (Table 5), as well as between TWSTRS disability and pain score and QST values. Only three of our patients reported neck pain. Therefore, the correlation of the pain subscore of the TWSTRS with QST values cannot be taken as significant.

**Table 5:** Correlations of Burke–Fahn–Marsden Dystonia Rating Scale (BFM) and Toronto Western Spasmodic Torticollis Rating Scale (TWSTRS) motor scores with Quantitative Sensory Testing values show no significant results.

	BFM		TWSTRS	
	Spearman	*p*-Value	Spearman	*p*-Value
CDT	−0.161	0.522	−0.186	0.489
HDT	−0.232	0.355	0.021	0.939
TSL	0.187	0.457	−0.132	0.939
CPT	−0.435	0.71	−0.429	0.097
HPT	0.084	0.741	0.379	0.148
TDT	0.186	0.459	−0.533	0.34
MPT	−0.164	0.543	−0.349	0.202
DMA	0.355	0.205	0.633	0.09
PSP	0.163	0.545	0.209	0.456
WUR	0.091	0.746	−0.528	0.098
VT	0.313	0.238	−0.507	0.1
PPT	0.258	0.374	0.127	0.679

*p-Value is considered significant when p < 0.05*.

*CDT, cold detection threshold; HDT, hot detection threshold; TSL, thermal sensory limen; CPT, cold pain threshold; HPT, hot pain threshold; TDT, tactile detection threshold; MPT, mechanical pain threshold; PSP, mechanical pain sensitivity for pinprick; DMA, dynamic mechanical allodynia; WUR, wind up ratio; VT, vibration threshold; PPT, pressure pain threshold*.

## Discussion

Our study shows that QST may detect subtle sensory abnormalities in patients with dystonia in absence of overt sensory deficits. Alterations of sensory function were found for modalities that are transmitted by C fibres (cold detection, partly cold pain threshold, and thermal sensory limen), by Aδ fibres (hot detection, cold pain threshold, thermal sensory limen) (23), and by Aβ fibres (dynamic mechanical allodynia) (24).

The main findings were decreased cold detection threshold, increased dynamic mechanical allodynia, and thermal sensory limen in the hand QST regardless of the distribution of dystonia. Our findings are partly in line with an earlier study, which investigated QST in a group of patients with idiopathic dystonia confined to the hand (25). While decreased cold detection threshold of the hand were found in both studies, we could not confirm abnormalities in MPTs and pain sensitivity for pinprick. Moreover, our patients showed increased dynamic mechanical allodynia in comparison to controls. Notably, the study of Suttrup and colleagues included only patients with writer’s cramp.

As demonstrated in our study, bilateral sensory alterations on both upper extremities were more predominant on the clinically more affected side. Interestingly, subtle sensory impairment was also detected on the hands of patients with cervical dystonia in our study. These findings suggest that clinically silent sensory alterations may be present in patients with dystonia regardless of the presence of overt dystonia in a specific body region. To our knowledge, shoulder QST has not been investigated previously in patients with cervical dystonia. The increase in dynamic mechanical allodynia and cold pain threshold compared to healthy controls suggest that this area may be suitable for further examination of the sensory system in patients with dystonia.

Dynamic mechanical allodynia (DMA) enhancement was the most consistent finding in our study. It is a stimulus evoked pain (26) that is induced by light touch (brush, cotton wool, and Q tip). Notably, our patients perceived such light stimuli as sharp or painful. The DMA test in the QST is designed to assess pain elicted by non-painful stimuli. All sensations that are perceived as sharp or painful (including those elicted by brush, cotton wool, and Q-tip) had to be scored higher than 0 on an analogue scale. In this point, the patients with dystonia showed most impairment in sensory perception. Patients with dystonia conceived altered perception of the quality of the stimuli (sharp instead of dull) but not a marked hyperalgesia. We refer to this sensory modality as DMA, as it is defined as such in the QST battery.

Moreover, allodynia for punctuate stimuli (pain sensitivity for pinprick) was not indicated by the subjects in our study. Therefore, pathophysiological mechanisms that have been proposed for DMA in peripheral and central neuropathic pain or in post stroke pain cannot explain the impairment of this sensory modality in dystonia.

Although we found that somatosensory processing is also impaired in clinically distant areas, the *z*-scores of the upper extremity more affected with dystonia showed a higher shift from the values of the controls than the upper extremity less affected by dystonia in all its domains. Remarkably, there was no correlation between severity of dystonia according to the motor scores (BFM, TWSTRS) and QST alterations. This may point to the presence of generalized subclinical sensory abnormalities in patients with dystonia independent from the site of the clinical manifestation of dystonia. Loss of sensitivity in temporal and spatial discrimination in patients with primary dystonia has been demonstrated before (11, 13, 15, 27) and in non-affected body regions as well (16, 28, 29). Abnormal spatial discrimination thresholds were found even in unaffected relatives (siblings and children) of patients with sporadic adult onset dystonia (10, 30). Moreover, abnormalities in temporal perception of consecutive stimuli have been shown in unaffected DYT1 carriers, which implies that sensory alterations in dystonia are not a mere consequence of abnormal movements, but may occur apart from motor manifestations (31). Furthermore, it is of interest that botulinum toxin or pallidal DBS did not appear to normalise temporal discrimination thresholds in patients with cervical dystonia, which means that alterations in temporal perception might be causal or related to the genesis of dystonia, rather than being an epiphenomenon secondary to abnormal motor activity (32, 33).

The pathomechanisms for the QST abnormalities seen in dystonia remain unclear. It has been suggested that sensorimotor integration plays a major role in the pathophysiology of dystonia (3). The basal ganglia are thought to have a role in the modulation of sensory stimuli and a direct impact on somatosensory integration (34–36). Furthermore, the cerebellum receives input from the spinal cord and interacts with the somatosensory system (37, 38). Functional imaging studies have revealed abnormalities in the basal ganglia circuitry as well as in the cerebellum in dystonia, even when lesions were not evident in structural imaging methods (7, 39–43).

Furthermore, altered neuroplasticity in the somatosensory cortex related to deranged somatotopic representation, such as abnormal cortical finger representation and disordered homuncular arrangement, was found in patients with focal dystonia (44–46), in some studies evident even bilaterally (47). Abnormal plasticity has been demonstrated in several types of dystonia (48), and it has been suggested to be a result of intrinsic defects in plasticity mechanisms or of the breakdown of such mechanisms (41). Notably, abnormal plasticity may lead to changes in the connectivity of the sensory and motor networks resulting in abnormal sensory and motor function.

Such complex functional changes in the brain of patients with dystonia may also underlie the sensory alterations we have detected with QST. Our findings may suggest that deranged somatosensory integration and abnormal plasticity can lead to the less reliable differentiation of somatosensory stimuli (dynamic mechanical allodynia, temperature thresholds) and a slower detection of certain sensations (thermal sensory limen). Whether these impairments are the consequence of a deficit of global sensorimotor integration or of the specific levels of the loop, or even a process of maladaptive plasticity itself, remains to be elucidated.

Moreover, it remains open to discuss whether abnormalities as observed in our study represent a primary sensory loss or a reaction to maladjusted central changes. Further analysis of dystonia may provide a better understanding of the sensory alterations in these patients. The limitations of this exploratory study are the small dystonia patient sample group that was tested with QST and the lack of correction for multiple comparisons. The forthcoming studies should focus on a larger number of patients, including specific inherited dystonias. Finally, future studies may address the specific role of the thalamo-striato-cortical and cerebello-thalamo-cortical pathways associated with the disturbed sensorimotor integration in dystonia.

## Ethics Statement

This study was carried out in accordance with the recommendations of Hannover Medical School with written informed consent from all subjects. All subjects gave written informed consent in accordance with the Declaration of Helsinki. The protocol was approved by the ethics committee of Hannover Medical School (No. 6307).

## Author Contributions

LP did the conception and design of the study, recruited subjects, collected data, performed statistical analysis, interpreted the data, and wrote the manuscript. FW concepted and designed the study, recruited the patients, collected and interpreted data, and contributed to the manuscript. LP and FW contributed equally. CB, MK, MA, AS, and DD recruited the patients, collected data, and approved the manuscript. JKK supervised the research project, concepted and designed the study, recruited patients, and contributed essentially to the manuscript.

## Conflict of Interest Statement

The authors declare that the research was conducted in the absence of any commercial or financial relationships that could be construed as a potential conflict of interest.

## References

[1] Albanese A Bhatia K Bressman SB Delong MR Fahn S Fung VS Phenomenology and classification of dystonia: a consensus update. Mov Disord (2013) 28:863–73. 10.1002/mds.25475 23649720 PMC3729880

[2] Jinnah HA Teller JK Galpern WR. Recent developments in dystonia. Curr Opin Neurol (2015) 28:400–5. 10.1097/WCO.0000000000000213 26110799 PMC4539941

[3] Quartarone A Hallett M. Emerging concepts in the physiological basis of dystonia. Mov Disord (2013) 28:958–67. 10.1002/mds.25532 23893452 PMC4159671

[4] Carbon M Kingsley PB Tang C Bressman S Eidelberg D. Microstructural white matter changes in primary torsion dystonia. Mov Disord (2008) 23:234–9. 10.1002/mds.21806 17999428 PMC4456010

[5] Hallett M Pathophysiology of dystonia. J Neural Transm (2006) 70:485–8. 10.1007/978-3-211-45295-0_72 17017571

[6] Sako W Fujita K Vo A Rucker JC Rizzo JR Niethammer M The visual perception of natural motion: abnormal task-related neural activity in DYT1 dystonia. Brain (2015) 138:3598–609. 10.1093/brain/awv282 26419798 PMC4840548

[7] Argyelan M Carbon M Niethammer M Ulug AM Voss HU Bressman SB Cerebello-thalamocortical connectivity regulates penetrance in dystonia. J Neurosci (2009) 29:9740–7. 10.1523/JNEUROSCI.2300-09.2009 19657027 PMC2745646

[8] Abbruzzese G Berardelli A. Sensorimotor integration in movement disorders. Mov Disord (2003) 18:231–40. 10.1002/mds.10327 12621626

[9] Berardelli A Rothwell JC Hallett M Thompson PD Manfredi M Marsden CD. The pathophysiology of primary dystonia. Brain (1998) 121:1195–212. 10.1093/brain/121.7.1195 9679773

[10] Walsh R O’Dwyer JP Sheikh IH O’Riordan S Lynch T Hutchinson M. Sporadic adult onset dystonia: sensory abnormalities as an endophenotype in unaffected relatives. J Neurol Neurosurg Psychiatry (2007) 78:980–3. 10.1136/jnnp.2006.105585 17702779 PMC2117873

[11] Bradley D Whelan R Kimmich O O’Riordan S Mulrooney N Brady P Temporal discrimination thresholds in adult-onset primary torsion dystonia: an analysis by task type and by dystonia phenotype. J Neurol (2012) 259:77–82. 10.1007/s00415-011-6125-7 21656045

[12] Kimmich O Molloy A Whelan R Williams L Bradley D Balsters J Temporal discrimination, a cervical dystonia endophenotype: penetrance and functional correlates. Mov Disord (2014) 29:804–11. 10.1002/mds.25822 24482092

[13] Hutchinson M Kimmich O Molloy A Whelan R Molloy F Lynch T The endophenotype and the phenotype: temporal discrimination and adult-onset dystonia. Mov Disord (2013) 28:1766–74. 10.1002/mds.25676 24108447

[14] Tinazzi M Fiorio M Fiaschi A Rothwell JC Bhatia KP. Sensory functions in dystonia: insights from behavioral studies. Mov Disord (2009) 24:1427–36. 10.1002/mds.22490 19306289

[15] Bara-Jimenez W Shelton P Hallett M. Spatial discrimination is abnormal in focal hand dystonia. Neurology (2000) 55:1869–73. 10.1212/WNL.55.12.1869 11134387

[16] Molloy FM Carr TD Zeuner KE Dambrosia JM Hallett M. Abnormalities of spatial discrimination in focal and generalized dystonia. Brain (2003) 126:2175–82. 10.1093/brain/awg219 12821512

[17] Rolke R Magerl W Campbell KA Schalber C Caspari S Birklein F Quantitative sensory testing: a comprehensive protocol for clinical trials. Eur J Pain (2006) 10:77–88. 10.1016/j.ejpain.2005.02.003 16291301

[18] Flor H Rasche D Islamian AP Rolko C Yilmaz P Ruppolt M Subtle sensory abnormalities detected by quantitative sensory testing in patients with trigeminal neuralgia. Pain Physician (2016) 19:507–18. 27676667

[19] Freund B Schwartz M. Temporal relationship of muscle weakness and pain reduction in subjects treated with botulinum toxin A. J Pain (2003) 4:159–65. 10.1054/jpai.2003.435 14622713

[20] Rolke R Baron R Maier C Tolle TR Treede RD Beyer A Quantitative sensory testing in the German Research Network on Neuropathic Pain (DFNS): standardized protocol and reference values. Pain (2006) 123:231–43. 10.1016/j.pain.2006.01.041 16697110

[21] Baumgaertner U Magerl W Klein T Hopf HC Treede RD. Neurogenic hyperalgesia versus painful hypoalgesia: two distinct mechanisms of neuropathic pain. Pain (2002) 96:141–51. 10.1016/S0304-3959(01)00438-9 11932070

[22] Herrero JF Laird JMA Lopez-Garcia JA. Wind-up of spinal cord neurones and pain sensation: much ado about something? Prog Neurobiol (2000) 61:169–203. 10.1016/S0301-0082(99)00051-9 10704997

[23] Beissner F Brandau A Henke C Felden L Baumgärtner U Treede RD Quick discrimination of A(delta) and C fiber mediated pain based on three verbal descriptors. PLoS One (2010) 5:e12944. 10.1371/journal.pone.0012944 20886070 PMC2944851

[24] Campbell JN Raja SN Meyer RA Mackinnon SE. Myelinated afferents signal the hyperalgesia associated with nerve injury. Pain (1988) 32:89–94. 10.1016/0304-3959(88)90027-9 3340426

[25] Suttrup I Oberdiek D Suttrup J Osada N Evers S Marziniak M. Loss of sensory function in patients with idiopathic hand dystonia. Mov Disord (2011) 26:107–13. 10.1002/mds.23425 20960475

[26] Sandkühler J. Models and mechanisms of hyperalgesia and allodynia. Physiol Rev (2009) 89:707–58. 10.1152/physrev.00025.2008 19342617

[27] Scontrini A Conte A Defazio G Fiorio M Fabbrini G Suppa A Somatosensory temporal discrimination in patients with primary focal dystonia. J Neurol Neurosurg Psychiatry (2009) 80:1315–9. 10.1136/jnnp.2009.178236 19541688

[28] Putzki N Stude P Konczak J Graf K Diener HC Maschke M. Kinesthesia is impaired in focal dystonia. Mov Disord (2006) 21:754–60. 10.1002/mds.20799 16482525

[29] Fiorio M Tinazzi M Scontrini A Stanzani C Gambarin M Fiaschi A Tactile temporal discrimination in patients with blepharospasm. J Neurol Neurosurg Psychiatry (2008) 79:796–8. 10.1136/jnnp.2007.131524 17986501

[30] Walsh RA Whelan R O’Dwyer J O’Riordan S Hutchinson S O’Laoide R Striatal morphology correlates with sensory abnormalities in unaffected relatives of cervical dystonia patients. J Neurol (2009) 256:1307–13. 10.1007/s00415-009-5119-1 19353218

[31] Fiorio M Gambarin M Valente EM Liberini P Loi M Cossu G Defective temporal processing of sensory stimuli in DYT1 mutation carriers: a new endophenotype of dystonia. Brain (2007) 130:134–42. 10.1093/brain/awl283 17105745

[32] Sadnicka A Kimmich O Pisarek C Ruge D Galea J Kassavetis P Pallidal stimulation for cervical dystonia does not correct abnormal temporal discrimination. Mov Disord (2013) 28:1874–7. 10.1002/mds.25581 23853089

[33] Scontrini A Conte A Fabbrini G Colosimo C Di Stasio F Ferrazzano G Somatosensory temporal discrimination tested in patients receiving botulinum toxin injection for cervical dystonia. Mov Disord (2011) 26(4):742–6. 10.1002/mds.23447 21506155

[34] Kaji R. Basal ganglia as a sensory gating devise for motor control. J Med Invest (2001) 48:142–6. 11694953

[35] Graybiel AM. Network-level neuroplasticity in cortico-basal ganglia pathways. Parkinsonism Relat Disord (2004) 10:293–6. 10.1016/j.parkreldis.2004.03.007 15196508

[36] Ding JB Guzman JN Peterson JD Goldberg JA Surmeier DJ. Thalamic gating of corticostriatal signaling by cholinergic interneurons. Neuron (2010) 67:294–307. 10.1016/j.neuron.2010.06.017 20670836 PMC4085694

[37] Oulad Ben Taib N Manto M Laute MA Brotchi J. The cerebellum modulates rodent cortical motor output after repetitive somatosensory stimulation. Neurosurgery (2005) 56:811–20. 10.1227/01.NEU.0000156616.94446.00 15792520

[38] Daskalakis ZJ Paradiso GO Christensen BK Fitzgerald PB Gunraj C Chen R. Exploring the connectivity between the cerebellum and motor cortex in humans. J Physiol (2004) 557:689–700. 10.1113/jphysiol.2003.059808 15047772 PMC1665103

[39] Lehéricy S Tijssen MA Vidailhet M Kaji R Meunier S. The anatomical basis of dystonia: current view using neuroimaging. Mov Disord (2013) 28:944–57. 10.1002/mds.25527 23893451

[40] Zoons E Booij J Nederveen AJ Dijk JM Tijssen MA Structural, functional and molecular imaging of the brain in primary focal dystonia a review. Neuroimage (2011) 56:1011–20. 10.1016/j.neuroimage.2011.02.045 21349339

[41] Neychev VK Gross R Lehericy S Hess EJ Jinnah HA. The functional neuroanatomy of dystonia. Neurobiol Dis (2011) 2011(42):185–201. 10.1016/j.nbd.2011.01.026 21303695 PMC3478782

[42] Niethammer M Carbon M Argyelan M Eidelberg D. Hereditary dystonia as a neurodevelopmental circuit disorder: Evidence from neuroimaging. Neurobiol Dis (2011) 2011(42):202–9. 10.1016/j.nbd.2010.10.010 20965251 PMC3062649

[43] Carbon M Argyelan M Eidelberg D. Functional imaging in hereditary dystonia. Eur J Neurol (2010) 17(Suppl 1):58–64. 10.1111/j.1468-1331.2010.03054.x 20590810 PMC4643651

[44] Catalan MJ Ishii K Bara-Jimenez W Hallett M. Reorganization of the human somatosensory cortex in hand dystonia. J Mov Disord (2012) 5:5–8. 10.14802/jmd.12002 24868405 PMC4027675

[45] Elbert T Candia V Altenmüller E Rau H Sterr A Rockstroh B Alteration of digital representations in somatosensory cortex in focal hand dystonia. Neuroreport (1998) 9:3571–5. 10.1097/00001756-199811160-00006 9858362

[46] Bara-Jimenez W Catalan MJ Hallett M Gerloff C. Abnormal somatosensory homunculus in dystonia of the hand. Ann Neurol (1998) 44:828–31. 10.1002/ana.410440520 9818942

[47] Meunier S Garnero L Ducorps A Mazières L Lehéricy S du Montcel ST Human brain mapping in dystonia reveals both endophenotypic traits and adaptive reorganization. Ann Neurol (2001) 50:521–7. 10.1002/ana.1234 11601503

[48] Quartarone A Rizzo V Morgante F. Clinical features of dystonia: a pathophysiological revisitation. Curr Opin Neurol (2008) 21:484–90. 10.1097/WCO.0b013e328307bf07 18607211

